# Assessment of biochemical bone markers of osteoporosis in children with thalassemia major

**DOI:** 10.1186/s13052-022-01290-x

**Published:** 2022-06-20

**Authors:** Tanju Çelik, Özlem Sangün, Şule Ünal, Ali Balcı, Sedat Motor

**Affiliations:** 1 School of Medicine, Pediatrics, Mustafa Kemal University, Hatay, Turkey; 2 Department of Pediatrics, University of Health Sciences Dr. Behçet Uz Children’s Hospital, İzmir, Turkey; 3 Department of Pediatric Endocrinology, Antakya State Hospital, Hatay, Turkey; 4 Department of Pediatric Hematology and Oncology, Antakya State Hospital, Hatay, Turkey; 5 Department of Pediatrics, Division of Hematology, Hacettepe University School of Medicine, Ankara, Turkey; 6 School of Medicine, Department of Radiology, 9 Eylul University, Hatay, Turkey; 7 School of Medicine, Department of Biochemistry, Mustafa Kemal University, Hatay, Turkey

**Keywords:** Bone markers, Bone mineralization disorders, Osteoporosis

## Abstract

**Background:**

Beta thalassemia major (β-TM) is a common cause of skeletal morbidity and is associated with increased bone fracture risk, particularly in inadequately transfused children. The aim of this study was to investigate some potential biochemical markers as possible early predictors of BMD variations in children with β-TM.

**Methods:**

The study included 38 children with β-TM and 40 sex-age matched controls. All patients were subjected to BMD assessment by dual-energy X-ray absorptiometry (DEXA). Serum beta-crosslaps (beta-CTx), osteoprotegerin (OPG), receptor activator of nuclear factor-kappa B ligand (RANKL), urinary deoxypyridinoline (DPD) and ferritin levels were compared between the groups.

**Results:**

Serum OPG levels were significantly lower in thalassemic children than in controls. The mean ratio of RANKL/OPG was significantly higher in the thalassemic patients than in the control group. Osteoporosis was detected in 10 (3 female and 7 male) of 38 patients (26.3%) according to the femur Z score and in 6 of them (4 male and 2 female) (15.8%) according to the spine Z score.

**Conclusions:**

Serum OPG concentrations can be used as a biochemical marker in screening patients with beta-thalassemia major for the development of osteoporosis.

## What is already known about this topic?

It is well known that **beta**-thalassemia major is associated with a number of comorbidities, including osteoporosis. Screening children with beta-thalassemia major for osteoporosis with dual-energy X-ray absorptiometry (DEXA) is necessary for detecting osteoporosis and improving bone health. However, DEXA is a radiological tool with X-ray exposure that is not available in every centre. Therefore, there is a need for biochemical markers.

## What does this study add?

This study shows that serum osteoprotegerin concentrations can be used as a biochemical marker in screening patients with beta-thalassemia major for the development of osteoporosis.

## Background

Beta thalassemia major (β-TM) is a common cause of skeletal morbidity and increased bone fracture risk in thalassemic patients [1]. Its pathogenesis is multifactorial and mainly includes bone marrow expansion, endocrine dysfunction and iron overload [2, 3]. The mechanisms through which these factors lead to bone loss have not been completely clarified [4]. Previous reports using dual-energy X-ray (DEXA) suggest that up to 70% of adults with β-TM have low bone mass [5]. The skeletal changes in thalassemic patients may include osteoporosis, growth failure, bone age delay and spondylometaphyseal abnormalities [6]. Some of the aetiological factors for thalassemic osteoporosis are dysfunction of the hypothalamic-pituitary-gonadal axis, growth hormone effects on the parathyroid gland, diabetes, hypothyroidism, ineffective haematopoiesis and direct toxicity of iron to osteoblasts. Iron chelation therapies may also cause osteopenia and osteoporosis via their effects on cartilage tissue [7, 8].

Osteoprotegerin (OPG) and the receptor activator of nuclear factor-kappa B (RANK)/receptor activator of nuclear factor-kappa B ligand (RANKL) are the major cytokines related to the regulation of bone resorption [9]. The receptor activator of the nuclear factor-kappa B (RANK)/RANK ligand (RANKL)/osteoprotegerin (OPG) pathway has been recently recognised as the final, dominant mediator of osteoclast proliferation and activation [8–11].

The aim of this study was to search for some potential biochemical markers [deoxypyridinoline (DPG), beta-crosslaps (β-CTX), OPG and RANKL]) as possible early predictors of BMD variations in children with β-TM.

## Materials and methods

### Study design

This was a case–control study that was conducted from 2011-to 2014. Ethical approval was obtained from the local ethics committee of Mustafa Kemal University with the decision number 2011/12. Written informed consent was obtained from all participants or if participants are under 16, from a parent and/or legal guardian. This study was supported by the division of the Scientific Research Projects of the institution.

The study included 38 children with β-TM who were on chronic transfusion therapy. The control group consisted of 40 patients admitted to the hospital for minor illnesses such as headaches or the routine management of a healthy child.

### Data analysis

Patients’ data files were revisited to determine to mean pretransfusion haemoglobin and mean serum ferritin levels in the year prior to the sample collection date of each patient. Blood and urine samples were studied at Ankara Duzen Laboratory (Turkey), which is accredited by the Turkish Accreditation Agency (TURKAK). The BMD values are compared with reference values from healthy children with similar age, sex, and ethnicity to calculate a Z score, the number of SDs from the expected mean. Z scores lower than − 2.0 were accepted as “low bone mineral density or bone mineral content for chronologic age”.

### Assessment of bone turnover markers

Five millilitres of fasting pre–transfusion venous blood was collected, and serum was stored at − 20 °C after separation. All children were asked to provide urine samples stored at − 20 °C until the laboratory assessment.

#### Deoxypyridinoline (DPD)

The DPD level was measured using the Chromsystem (HPLC). DPD is a marker of osteoclastic activity and bone resorption. Levels of urine deoxypyridinoline in urine do not vary with dietary intake; however, for consistency, we use second-morning urine (from 10 to 12 o’clock).

#### C-terminal telopeptide of collagen type-I (beta-crosslaps)

Beta-crosslaps were measured via the electrochemiluminescence method using the Cobas® kit (Roche, Germany) in the Cobas e601® analyser (Roche, Germany).

#### Receptor activator of nuclear factor-kappa B ligand (RANKL) and osteoprotegerin (OPG)

Serum osteoprotegerin was measured by a commercially available kit (Biovendor ELISA Biotec Synergy HT) using the BioVendor Human Osteoprotegerin ELISA. The intra-assay coefficient of variation was 4.5%, and the interassay variation was 7.8%, as provided by the manufacturer. Serum RANKL was measured by a commercially available kit (Biovendor ELISA Biotec Synergy HT) using the BioVendor Human sRANKL ELISA technique. The intra-assay coefficient of variation was 8.8%, and the interassay variation was 11%, as provided by the manufacturer.

### Statistical analysis

The Statistical Package for the Social Sciences (SPSS) 15.0 programme was used for statistical analysis. The Mann–Whitney U test and paired-sample t tests were applied to evaluate the differences between patients and controls. At the same time, the Wilcoxon signed-rank test was used to evaluate differences between baseline and values of the studied parameters at various time points. The correlation between changes in various biochemical parameters and BMD were evaluated with Spearman’s (rs) correlation coefficient. Variables were found to be statistically significant at *p* < 0.05.

## Results

The study group included 38 β-TM patients (13 female, 25 male) and 40 age- and sex-matched controls (19 female, 21 male). The mean age of the β-TM group was 12.9 ± 3 (9–22) years, while it was 12.5 ± 3 (6–18) years in the control group. The mean age and sex distribution of the groups were not significantly different (*p* > 0.05). Serum AST, ALT, BUN, creatinine, calcium, phosphorus, parathyroid hormone and 25-OH-D vitamin levels were within normal limits and did not differ between the patient and control groups.

Comparisons of major cytokines related to the regulation of bone resorption between patients with thalassemia major and healthy controls are presented in Table 1. Serum OPG concentrations were significantly lower in thalassemic children than in controls. The mean ratio of RANKL/OPG was significantly increased in children with beta-thalassemia compared to the control group (Table 1).

**Table 1:** Comparisons major cytokines related with regulation the bone resorption between patients with Thalassemia major and healthy controls

	β -TM group (***n*** = 38)	Control group (***n*** = 40)	P
**Age (y)** (mean ± SD)	12.92 ± 3.10	12.50 ± 3.12	t = − 0.596 *p* = 0.553
**DPD (**nM/mM Kr**)** (mean ± SD)	44.57 ± 24.14	51.29 ± 31.06	t = 1.069 *p* = 0.289
**Beta-CT**_**x**_ **(**pg/mL**)** (mean ± SD)	1025.76 ± 439.71	968.00 ± 388.46	t = − 0.614 *P* = 0.541
**OPG (**pmol/L**)** (mean ± SD)	2.01 ± 0.66	2.43 ± 0.57	t = 3.027 ***p*** **= 0.003**
**RANKL (**pmol/L**)** (mean ± SD)	346.18 ± 142.63	318.05 ± 110.75	t = − 0.976 *p* = 0.332
**RANKL/OPG**	46.09	33.24	***p*** **= 0.012**

*DPD* Deoxypyridinoline, *OPG* Osteoprotegerin, *RANKL* Receptor activator of NF-κB ligand, *β –TM* Beta-thalassemia, *Beta-CTx* Beta-Crosslaps

In this study, the values of DPD, OPG, beta-CTX and RANKL, DEXA, FSH, LH, oestradiol, vitamin D3, TSH, T4, dehydroepiandrosterone (DHEA-SO4), testosterone, AST, ALT, BUN, creatinine, calcium, phosphorus, alkaline phosphatase, parathormone, total protein, albumin, ferritin, white blood cells, haemoglobin and platelets were compared between the groups. There were significant negative correlations between the OPG value and DEXA, PTH and phosphorus. PTH/phosphorus ratio. (r: 0.55, p: 0.003) (phosphorus: r: 0.38, p: 0.029). There was a significantly positive correlation between RANKL and AST, and ALT. There was a significantly negative correlation between the DPD value and TSH and T4 levels. There was a significantly positive correlation between the beta-CTX value and alkaline phosphates (Table 2). There were no significant correlations among the other parameters (*p* > 0.05).

**Table 2:** Correlations DEXA, DPD, Beta CTx, OPG, RANK and biochemical markers in Thalassemia major patients

	DEXA	DPD	Beta-CT_**x**_	OPG	RANKL
Age	**.480**^**⁎⁎**^	0,38	0,37	0,38	0,38
DEXA	0,38	0,23	**-0,34**^**⁎**^	**−.412**^**⁎**^	**0,36**^**⁎**^
PTH	**− 0,40**^**⁎**^	0,17	0,079	**-,557**^**⁎**^	−0,21
Phosphorus	0,29	0,038	−0,18	**,380**^**⁎**^	0,28
TSH	**−0,55**^**⁎**^	**,397**^**⁎**^	0,23	0,06	0,03
T4	**−0,56**^**⁎**^	**,458**^**⁎**^	0,08	0,19	0,05
AST	−0,22	− 0,07	−0,16	0,05	**,373**^**⁎**^
ALT	−0,22	0,05	−0,18	0,10	**,346**^**⁎**^
Alkaline Phosphatase	−0,24	0,24	**,383**^**⁎**^	0,08	−0,007
Ferritin	**−0.38**^**⁎**^	0,07	0,02	0,05	0,22

* *p* < 0.05, ** *p* < 0.01

Femur and spine Z scores of the thalassemic patients were evaluated with DEXA. The mean value of the lumbar spine Z score was − 1.81 ± 0.93 (median − 1.81), and the femur Z score was − 1.6 ± 0.99 (median-1.60) in the thalassemic patients. Lumbar site osteopenia was present in 36.4% of the patients (12/33), and 33.3% (11/33) of them had osteopenia at the femur site. Osteoporosis was detected in 10 (3 female and 7 male) of 38 patients (26.3%) according to the femur Z score and in 6 of them (4 male and 2 female) (15.8%) according to the spine Z score.

When the groups were compared according to sex, the mean spine DEXA Z score results were higher in females than males (− 1.71 ± 0.86 SD versus − 1.89 ± 0.89 SD), while the mean femur Z score was higher in males than females (− 1.59 ± 0.99 SD versus − 1.74 ± 0.85). The spine Z score was negatively correlated with age and ferritin levels (r: 0.484 p: 0.002 for age and r: 0.380 p: 0.02 for ferritin).

Regression analysis was performed to assess the factors affecting the spine Z score. Age and ferritin levels significantly affected the spine Z score. The spine Z score was determined with Dubin Watson regression analyses with the formula “spine Z score = (− 0.125x age) + (9.4x ferritin level)”. According to this formula, the Z score declined as age and ferritin levels increased.

## Discussion

Beta-thalassemia major (β-TM) is a common cause of skeletal morbidity and increased bone fracture risk in thalassemic patients^1^. Its pathogenesis is multifactorial and mainly includes bone marrow expansion, endocrine dysfunction and iron overload [2, 3]. In this study, we investigated the DPD, beta-CTX, OPG and RANKL values of our patients diagnosed with β-TM. We compared these values with DEXA and hormonal changes to observe whether there was a correlation between them.

OPG levels were significantly decreased in thalassemic patients, although no significant difference was detected between the serum RANKL levels of the groups. The most important result of this finding is an increased RANKL/OPG ratio in thalassemic children. Morabito et al. [10] reported that thalassemic patients showed no differences in plasma levels of OPG compared with controls and significantly higher plasma levels of RANKL, with a consequent significantly lower OPG/RANKL ratio (which is equal to a higher RANKL/OPG ratio) in an adult study. These results indicate that low bone mass can be associated with decreased osteoblastic activity in children despite increased osteoclastic activity in adults [10]. Our findings are correlated with Morabito’s study. Ozdemir et al. [11] reported that vitamin menaquinone-7 and calcitriol have beneficial effects on thalassemic osteopathy.

Alfaqih et al. [12] reported that plasma RANKL level was the most significant marker for bone resorption and strongly correlated with the N-terminal propeptide of type 1 collagen; lower in individuals with thalassemia intermedia.

Osteoblasts and osteoclasts are responsible for bone remodelling at the cellular level. In adult thalassemic patients, RANK/RANKL-OPG system disorder increases in favour of osteoclasts. Many studies have noted that the RANKL/OPG ratio increases in patients with thalassemia major, and this situation shows the role of this system in the pathogenesis of osteoporosis [13–15]. Toumba and Skordis [6] reported that in thalassemia patients, progressive “ageing” of the bone starts even in childhood by the gradual development of an imbalance between augmented osteoclastic resorption and insufficient osteoblastic bone formation. We suggest that osteoblastic dysfunction is the dominant mechanism of osteopenia in prepubertal children.

We did not find any association with sex steroids, OPG, RANKL and DEXA, which agrees with different studies from Italy and Greece [16, 17].

Several studies have shown that PTH acts by enhancing the production of RANKL and by inhibiting the synthesis of OPG [18, 19]. However, we detected a positive correlation between PTH and OPG levels in this study. These results may be attributed to the unique characteristics of the growing bones and the high rate of bone turnover in childhood. There were no other significant correlations between the markers of bone turnover and PTH in this study.

In a study of 42 patients diagnosed with thalassemia major, the rate of osteoporosis by DEXA was 81%. β-CTX and PTH levels were high, but vitamin D levels were low in the same group [16]. This study similarly shows high levels of PTH and alkaline phosphatase and an increased ratio of RANKL/OPG in the thalassemic group. Although the difference was not statistically significant, the mean β-CTX levels of the thalassemic patients were higher than those in our study’s control group. These biochemical markers serve as a signal in the development of osteoporosis in patients with beta-thalassemia major.

It is not clear whether sex differences influence bone diseases [20]. Some studies report that sex differences have a role in developing osteoporosis syndrome in thalassemia and affect its severity. Males are more frequently and severely affected by osteopenia and osteoporosis than females, and some others report that sex differences are not significant [20, 21]. In a study conducted on patients with sickle cell anaemia, the mean lumbar spine Z score was significantly lower in prepubertal girls than in boys. However, there was no significant difference in the pubertal group [22]. When the prepubertal thalassemic patients in this study were evaluated according to sex, the mean spine DEXA Z score results were higher in females than males. In contrast, the mean femur Z score was higher in males than females.

Iron accumulation has a negative effect on bone mineralisation. BMD was significantly influenced by noneffective chelation in children with beta-thalassemia major [23]. According to the results of this study, it was confirmed that DEXA Z scores similarly decreased with increasing levels of ferritin.

### Limitations

One of the most important limitations of the study is that the participants were not homogeneous in terms of the duration of thalassemia disease. This may affect the degree of osteoporosis in these patients, and thus the correlation between osteoporosis and the potential markers studied. The inability to perform DEXA examination in healthy controls is another important limitation. The possibility of osteoporosis among these patients may confound the comparisons between patients with β-TM and healthy controls. Therefore, there is a need for larger-scale studies comparing the results of a more homogeneous study group in terms of disease duration and a control group that is proven to be free of osteoporosis.

## Conclusion

Osteoporosis is a multifactorial disease and may occur early, especially in chronic diseases such as thalassemia. Because of the difficulties in diagnosis and follow-up, screening with DEXA and measuring ferritin level and RANKL/OPG ratios on a regular basis is essential. It should be kept in mind that osteoporosis may develop with advancing age in both sexes.

## Data Availability

The datasets used and/or analyzed during the current study are available from the corresponding author on reasonable request.

## Abbreviations

β-CTX: Beta-crosslaps
β-TM: Beta Thalassemia Major
DPD: Deoxypyridinoline
DEXA: Dual-Energy X-ray
RANKL: Activator of nuclear factor-kappa B ligand
OPG: Osteoprotegerin
TURKAK: Turkish Accreditation Agency
